# EARLY ONSET AGGRESSIVE BEHAVIOR INDUCED BY PERAMPANEL IN THE TREATMENT OF CHRONIC INSOMNIA: A CASE REPORT

**DOI:** 10.1192/j.eurpsy.2023.2343

**Published:** 2023-07-19

**Authors:** I. Esteban-Avendaño, J. Torres Cortés, J. Padín Calo

**Affiliations:** Department of Psychiatry, Hospital Universitario Ramón y Cajal, Madrid, Spain

## Abstract

**Introduction:**

Chronic insomnia, resistant to different treatments (pharmacological, sleep hygiene and cognitive-behavioral therapy) remains one of the greatest challenges in our daily practice as psychiatrists. The pharmacological options include benzodiazepines and their analogues (zolpidem, zopiclone, etc.). However, when trying to treat chronic insomnia the use of off-label drugs, including antidepressants with sedative action (such as trazodone), antipsychotics or antiepiletic drugs, is not uncommon.

Perampanel is a non-competitive AMPA receptor antagonist, marketed for the treatment of partial onset epilepsy and primary generalized tonic-clonic seizures. It has been used in the treatment of chronic insomnia with positive results and it has shown to improve the quality of sleep in a recent observational retrospective cohort study.

The most frequent adverse effects of Perampanel include dizziness and drowsiness. Perampanel can also cause psychiatric and behavioral adverse effects, aggression and irritability in up to 10% of patients, as well as depression, and suicidal ideation, with higher rates in patients with psychiatric history.

**Objectives:**

To draw attention to possible adverse effects of Perampanel and to add knowledge to improve the treatment for chronic insomnia.

**Methods:**

Case report and non-systematic literature review of the current data.

**Results:**

A 33 year old woman with Anorexia Nervosa was admitted to the psychiatric hospitalization unit due to suicidal ideation and a history of chronic insomnia. Perampanel was started at a dose of 2mg/day, progressively titrated to 6mg/day, following patient’s informed consent. A week after the initiation of treatment, her sleep pattern had improved but she became aggressive, showed low tolerability to minor frustrations and suffered from an intensification of suicidal ideation. She became extremely hostile to the personnel, had severe tantrums and deliberate self injurious behavior. Perampanel was discontinued and in less than a week her aggressive behavior succumbed. Although she was not re-exposed to Perampanel the symptoms she presented are considered a very likely adverse drug reaction. Levomepromazine 20mg/day and Lormetazepam 0.5mg/day were reinstated as a treatment for insomnia.

**Conclusions:**

Psychiatric comorbidity is known to be a risk factor for behavioral adverse effects of Perampanel. Therefore Perampanel as a treatment for chronic insomnia needs a careful individual benefit-risk assessment and monitoring for adverse effects.

**Disclosure of Interest:**

None Declared

